# Knowledge, attitude and practice in high endemic settings for malaria: a cross-sectional study in a rural community in central Mozambique

**DOI:** 10.1186/s12936-025-05652-8

**Published:** 2025-11-28

**Authors:** Joaquim Domingos Lequechane, Falume Chale, Nicole Azevedo, Ana Paula Abílio, Alberto Gabriel Muanido, Luzia Gonçalves, João Luís Manuel, Henrique Silveira

**Affiliations:** 1 Global Health and Tropical Medicine (GHTM), Associate Laboratory in Translation and Innovation Towards Global Health (LA-REAL), Instituto de Higiene e Medicina Tropical, Universidade Nova de Lisboa (IHMT-NOVA), Rua da Junqueira 100, 1349-008 Lisbon, Portugal; 2 Centro de Investigação Operacional da Beira (CIOB), Instituto Nacional de Saúde, Ministério da Saúde, Rua Correia de Brito 1323, Ponta Gêa, Cidade da Beira, Mozambique; 3 Fundação Belmiro de Azevedo, Praça de Liège, 146, 4150-455 Porto, Portugal; 4 Instituto Nacional de Saúde de Moçambique, Vila de Marracuene, Maputo, Mozambique; 5 Centro de Estatística E Aplicações da Universidade de Lisboa (CEAUL), Campo Grande, Lisbon, Portugal; 6 Z-Stat4life, Espaço Cowork Baldaya, Palácio Baldaya, Estrada de Benfica Nº701A, 1549-011 Lisbon, Portugal; 7 Comité Para Saúde de Moçambique (CSM), Beira, Mozambique

**Keywords:** Malaria, Knowledge, Attitude, Practice, Spatial repellent, Mozambique

## Abstract

**Background:**

Malaria remains a public health threat globally, mainly in African countries. Despite the progress made over the years, its control and elimination by 2030 appear to be a challenge. The community perspective on factors contributing to malaria burden is essential to develop preventive measures adapted to local contexts. Understanding the transmission rates, symptoms, prevention and treatment of malaria are essential for a population to effectively control the disease. This study assessed the knowledge, attitude, and practice (KAP) of Tambai residents to identify the possible gaps related to high malaria burden in a study implementation area.

**Methods:**

This was a cross-sectional study design with a qualitative approach held in Sofala province, Nhamatanda district, Tambai community. This KAP study was carried out before the intervention in May 2021 and after in June 2022. All heads of households from the study implementation area were surveyed using a semi-structured questionnaire. Data were processed in Microsoft excel, exported and analysed in Statistical Packages for the Social Sciences (SPSS) version 29.0.

**Results:**

The knowledge of the heads of households on symptoms, mode of transmission, prevention and treatment of malaria was high before and after the intervention (149; 96.8%) and (143; 91.1%), respectively. Heads of households show no differences on KAP regarding symptoms, mode of transmission, prevention and treatment of malaria when intervention and control or before and after intervention were compared.

**Conclusion:**

Both intervention and control groups showed high KAP before and after intervention, suggesting that the implementation of the REPELMALARIA project did not have an either positive nor negative impact on KAP scores. These findings suggests that the higher malaria cases in the study area are not related to the malaria knowledge, attitude and practice of householders.

Trial registration: This results are from the main clinical trial registed in ClinicalTrials.gov ID: NCT04419766.

**Supplementary Information:**

The online version contains supplementary material available at 10.1186/s12936-025-05652-8.

## Background

Malaria remains a major public health issue, and it´s elimination by 2030 is a challenge, despite the considerable progress made in its control. In 2023 an estimated 263 million malaria cases and 597 000 deaths were reported worldwide. Sub-Saharan Africa accounted for 94% of the cases and 95% of deaths from the disease [1]. Mozambique is one of the five countries with the highest number of malaria cases and deaths worldwide [2]. *Plasmodium falciparum* is the most prevalent species and accounted for the most severe malaria cases [3]. According to the 2022 Annual Report form the Mozambican Malaria Programme [4], 3,038,933 individuals were tested for malaria in Sofala province, of whom 1,446,234 (48.0%) tested positive. Nhamatanda district contributed with 15% of cases and 22% of deaths that occurred in the province. The Mutondo health area, that serves Tambai, reported testing 14,432 patients, of which 8870 (61.2%) were diagnosed with malaria.

In Mozambique, 66.7% of the population lives in rural areas [5], where most malaria cases are concentrated. These areas often have limited resources and difficult access. Moreover, climate changes and environmental conditions are determinant factors for persistence and spread of malaria vectors and other vector borne diseases [6]. A nationwide mass distribution campaign of insecticide-treated nets targeting almost all population, takes place every 2–3 years in the country. Additionally, interventions aiming at raising awareness about malaria prevention, including social media campaigns, training for community leaders, distribution of pamphlets, and lectures in health facilities and communities are regularly performed [7].

The community perspective on factors associated with malaria burden is fundamental for developing preventive measures tailored to the local reality [8]. Furthermore, understanding transmission mode, symptoms, prevention and treatment are essential for a population to effectively prevent and control the disease. Knowledge, attitude and practice (KAP) studies have proven to be important tools to identify gaps in community understanding regarding malaria prevention and control [9]. The local knowledge can support provincial and national health authorities to design adequate malaria control interventions, tailored to regional needs [10].

A pre- and post-intervention KAP study conducted in Dakar, Senegal assessed the impact of school children’s awareness of malaria using the MOSKI KIT^®^ tool. The study showed an increase in knowledge among children in the intervention group during year 1, though no significant difference was observed when compared to school children in the control group [11]. In rural areas of central Mozambique, a cross-sectional KAP study on malaria prevention knowledge among women of reproductive age revealed a low malaria knowledge [12]. Another cross-sectional community-based KAP study, conducted in rural Ethiopia, in the Afar region, assessed knowledge, attitudes, and practice related to malaria prevention. The study revealed that while most heads of households had good knowledge of malaria prevention, some gaps in understanding were still observed among others [13]. There are further examples of KAP studies in other African countries showing good knowledge among the participants [14].

Ongoing strategies to control the disease, such as the distribution of sulfadoxine and pyrimethamine to pregnant women, integrated vector surveillance [15], and early diagnosis and treatment of all malaria cases [16], are actively being implemented. Despite the widespread implementation of these strategies or research trials that involve high exposure of the target population to specialized information, their impact on the knowledge, attitudes, and practices of target populations remains largely unassessed. Research has shown that knowledge of malaria transmission, prevention methods and treatment does not always translate into behavior change [17], highlighting the importance of evaluating how health interventions influence local attitudes and practices [18]. Further evaluations of these intervention´s impact on KAP are needed to assess their effectiveness in improving health outcomes and to inform future malaria control efforts.

While the Tambai community reports the highest malaria cases in Nhamatanda district, the reasons for this high malaria burden remain unclear. Therefore, this study assessed the knowledge, attitude, and practice of Tambai residents to identify possible gaps related to high malaria burden in a study implementation area.

## Methods

### Study settings

**Fig. 1:**
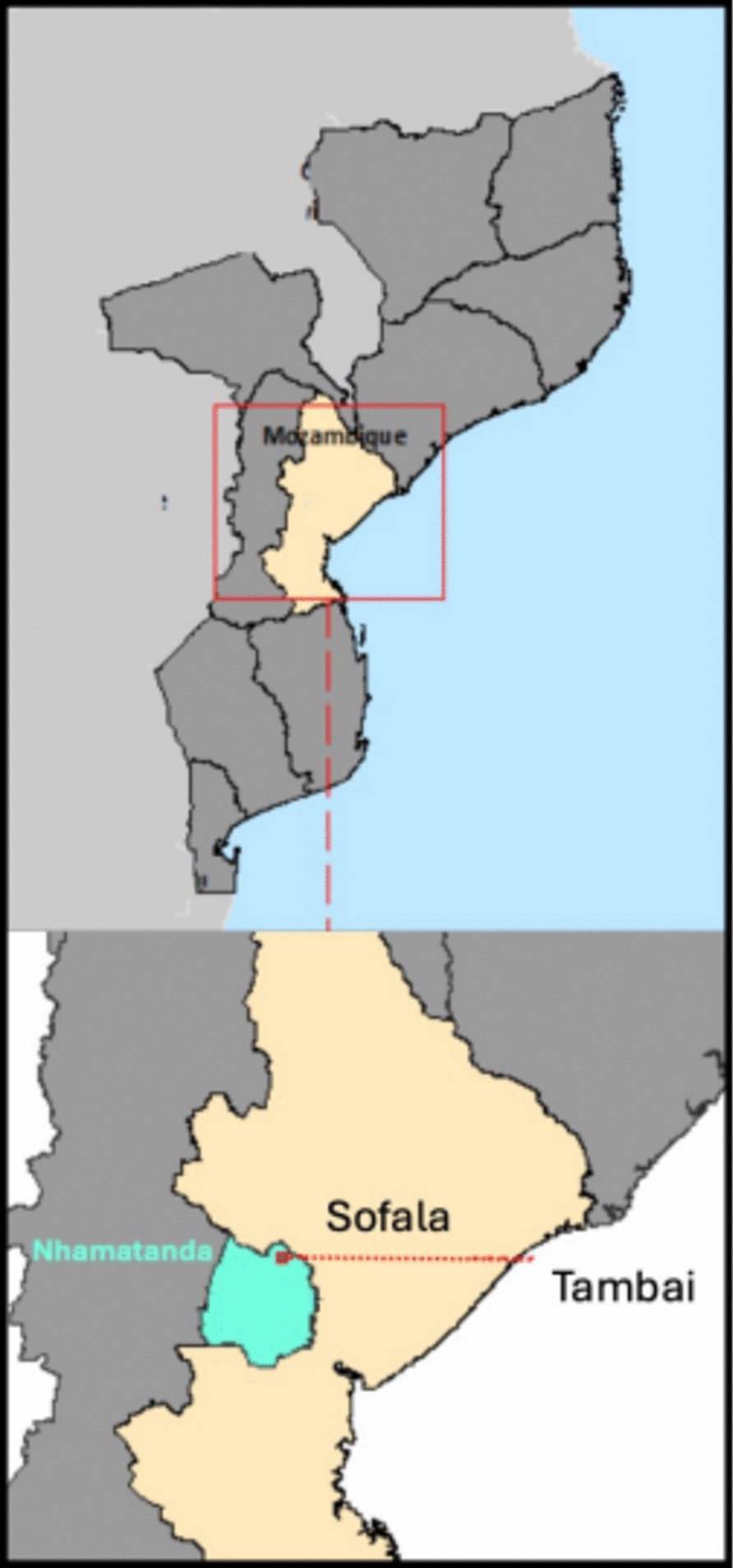
Map of Mozambique showing relative position of Tambai, Nhamatanda district, Sofala province where the study area was located

The study was conducted in the Tambai community, located in Nhamatanda district, Sofala province, in Mozambique, an area impacted by high malaria burden. Tambai is a remote community, located in the buffer zone of Gorongosa National Park. The region is swampy and has limited resources and lack of infrastructures, which makes access difficult, particularly during the rainy season. It is about 30 kms from the main village and falls under the jurisdiction of Mutondo Health Center. The community has approximately 1410 inhabitants and 157 householders. The climate is characterized by one hot and humid season from October to March and a dry and cool season from April to September. The study population consisted of all households from the study area (Fig. 1).

### Study design

This was a cross-sectional study with a quantitative approach aimed to evaluate the knowledge, attitude and practice (KAP) of heads of households from Tambai community, carried out in 2021 and 2022. These KAP surveys were part of the field trial entitled “Evaluation of *3-(n-acetyl-n-butyl) aminopropionic acid ethyl ester (IR3535)* as a spatial repellent for malaria control (REPELMALARIA) (ClinicalTrials.gov ID: NCT04419766), performed at Tambai, Nhamatanda district, Sofala province (Fig. 1). The first KAP survey was carried out from 27-05-2021 to 05–06-2021 and the second KAP survey was held from 28-05-2022 to 06-06-22, 1 year later. The REPELMALARIA project had intervention group (sprayed with mosquito repellent indoor and outdoor the houses) and control group (without spraying), on which the KAP study took place. Given the small population of households in the study area, all households were considered to participate in the study. Before intervention 154 heads of households consented to be interviewed, and 157 heads of households were interviewed after the intervention.

### Data collection

A week before data collection, a pre-test of data collection tool was performed with heads of households in the community of Mafambisse, Dondo District. The pre-test aimed to ensure the effectiveness, reliability and identify possibles problems with the data collection tool before starting the data collection. The pre-test was also a field training exercise for collectors. The observations from the pre-test were used to improve the data collection tool and practice.

Data were collected by two experienced and trained data collectors, using a semi-structured questionnaire based on Open Data Kit (ODK Collect), that enables data collection, using Android mobile devices. The survey was divided into sections, including sociodemographic and household characterization, ownership and use of mosquito nets, and knowledge, attitudes and practice (KAP), regarding cause, symptoms, mode of transmission and malaria prevention.

### Data analysis

Data were processed in Microsoft excel, then exported, coded and analysed in Statistical Packages for the Social Sciences (SPSS) version 29.0. Categorical variables were presented as frequencies and percentages, while continuous variables were presented as mean with standard deviation or median and interquartile range.

Since the information provided by the project team to a specific group and the entire population during the study implementation period could impact KAP of the households, KAP results were compared between intervention and control groups to assess the repellent effect. As general whole community engagement activities were performed throughout the project, a before-after comparison was also performed.

The results of KAP studies were initially analysed as dichotomic variables, where the knowledge had three categories (low, midle and high knowledge), attitude and practice had two categories (positive attitude/negative attitude and good/poor practice). Although dichotomizing of knowledge, attitude and practice facilitate the analysis, presentation and interpretation of results, the procedure can lead to loss of information. For this reason, the analysis considering the KAP scores were also carried out as continuous variables in order to overcome the loss of information [19, 20].

Chi-square (χ2) or Fisher exact test were used to assess association between knowledge, attitude and practice variables and sociodemographic or household characteristics, as well as to compare KAP between the intervention and control groups in 2021 and 2022. The Mann–Whitney or Kruskal–Wallis tests were used to evaluate association between KAP-scores and sociodemographic or household characteristics, and to compare KAP scores between intervention and control in 2021 and 2022. The Mann–Whitney test was used for independent variables with two categories, while the Kruskal–Wallis test was used for those with more than two categories.

To evaluate the knowledge the heads of households 5 questions were asked such as having heard of malaria; malaria symptoms; mode of transmission; prevention and treatment. Answers were considered correct if positive for having heard of malaria, they mentioned at least fever and headache as malaria symptoms, mosquito bite as mode of transmission, the use of mosquito net as preventive measure and Coartem as malaria treatment. Each correct answer was scored 1, while incorrect answer was scored 0. The knowledge score´s was categorized as: Low 0–2 scores (< 50%)], Medium 3 scores (50%−74%)] and High 4–5 scores (≥ 75%)[21].

To evaluate the attitudes, the householders were asked 4 questions: if they were at risk of getting malaria; own mosquito bed nets, actions when someone has a suspicion of malaria and if malaria is a fatal disease. Answers were considered correct if mentioned being at risk of getting malaria; owned mosquito bed net, take individuals with suspected malaria to the health facility; and were aware that malaria can be fatal. Each correct answer was scored 1 and incorrect answer were scored 0. The median score was calculated and used as cutoff. Heads of households with a score below the median (4.0) were classified as having negative attitudes, while those with the score equal to or above the median were classified as having positive attitudes.

To evaluate the practices, the heads of households were asked 3 questions related to whether they slept under a mosquito net in the previous night, how do they prevent malaria, and how do they respond when someone has malaria in the community. Answers were considered correct if they have slept under mosquito net in the previous night, use mosquito net to prevent against malaria and go to the health facility when someone has malaria. Each correct answer was scored 1 and incorrect answer were scored 0. The median score was calculated and used as cutoff. Heads of households with a score below the median (2.0) were classified as having poor practice, while those with the score equal to or above the median were classified as having good practices [22].

Variables with p ≤ 0.25 in the bivariate analysis were considered to binary and multinomial logistic regression [13]. The multinomial logistic regression was used to verify association of knowledge with sociodemografic and households characteristics. Binary regression was used to verify association of attitude and practice with sociodemografic and households characteristics. To verify the knowledge, attitude and practices associated factors the odds ratios (OR) and the confidence intervals (95% CI) were performed. Statistically significant was considered for p < 0.05.

### Ethical considerations

The study was approved by National Bioethics Committee for Health from Mozambique (ref.63/CNBS/20) and the last renewal letter is from August 20th, 2024 (512/CNBS/24). All participants were included by signing informed consent forms before starting the interview. For household heads who could not read and write, an impartial witness was recruited. The study followed all the ethical guidelines for research on human beings.

## Results

### Sociodemographic characteristics

A total of 154 and 157 heads of households were interviewed in 2021 and 2022, respectively. In 2021 the median age of heads of households was 33.5 (IQR: 24.75–50.50) years old. More than half were man [53.9% (83)], with primary school education [66.9% (103)], and married/marital union [76.0% (117)]. The main source of income was agriculture [90.9% (140)]. In 2022, from a total of 157 heads of households interviewed, 51.0% (80) were female, with primary school education 58.6% (92). The median age were 37.5 years old (IQR: 27–52) and agriculture was the main source of income [100.0% (157)].

### Household characteristics

In 2021, before the intervention, each household was composed by an average of 5 members (IQR: 3–7). All households [100.0% (154)] had at least a mosquito bed net and an average of two mosquito nets (IQR: 1.75–3.0). The majority of householders owned a mobile phone [59.1% (91)], bicycle [59.7% (92)] and animals [73.4% (113)]. The main water source was a standpipe [78.6% (121)]. Firewood was the power source used to prepare meals 100.0% (154). In 2022, the households were composed by a median of 5 members (IQR: 3.0–7.0), 94.9% (149) of households had mosquito nets, 58.6% (92) owned mobile phones, 59.9% (94) owned bicycle and 80.3% (126) owned animals at home. The main water source was a standpipe [63.7% (100)] and firewood was the power source used to prepare meals in all households 100.0% (157).

### Knowledge regarding symptoms, mode of transmission, prevention and treatment of malaria

In 2021, before the starting of the project, the knowledge of the heads of households regarding symptoms, mode of transmission, prevention and treatment of malaria was 96.8% (149). The majority of the respondents have heard of malaria 98.1% (151), recognized fever and headache as symptoms of malaria [94.0% (142)], mentioned mosquito bite as the malaria mode of transmission [100.0% (151)], knew that sleeping under the mosquito net can prevent against malaria [96.0% (145)] and recognize artemether-lumefantrine as malaria treatment [87.4% (132)]. In 2022, after one year of intervention, the knowledge of symptoms, mode of transmission, prevention and treatment of malaria was 91.1% (143). The majority has heard of malaria [94.9% (149)], mentioned fever and headache as the main symptoms of malaria[100.0% (149)], recognized the mosquito bite as malaria mode of transmission [96.0% (143)], pointed sleeping under the mosquito net as preventive measure of malaria [89.9% (134)] and 93.3% (139) mentioned artemether-lumefantrine as malaria treatment.

**Fig. 2:**
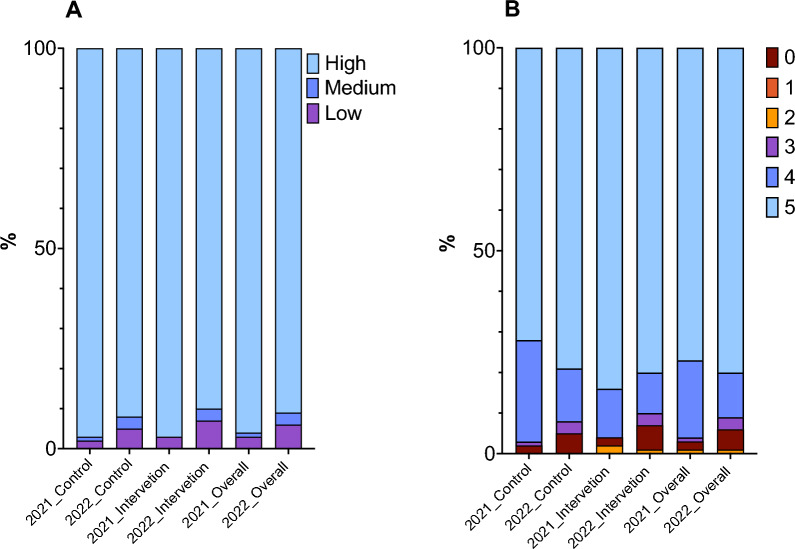
Knowledge of malaria symptoms, transmission, prevention, and treatment, and changes following intervention. **A** Categorized knowledge levels. The light blue bars represent high knowledge, the dark blue bar represents medium knowledge, and the purple bars represent low knowledge. High knowledge corresponds to the number of heads of households with equal or more than 75% correct answer; Medium knowledge correspond to 50%–74% of correct answers and low knowledge corresponds to less than 50% of correct answers. **B** Knowledge scores. The brownish red represents the number of participants with no scores, the yellow colour represents the number of participants with 2 scores, the dark pink represents the number of participants with 3 scores, the dark blue represents the number of participants with 4 scores, and the light blue represents the number of participants with 5 scores. Each score represents the number of correct answers, with higher scores indicating greater knowledge of malaria

The knowledge regarding malaria symptoms, transmission, prevention and treatment was higher in intervention and control in 2021 [96.9% (62), 96.7% (87)] when compared to 2022 [90.0% (63), 92.0% (80)], respectively (Fig. 2A). Overall knowledge score was equally high. More than 3/4 of the householders had the maximum knowledge score in 2021 [77.3% (119)] and in 2022 [79.6% (125)]. Among householders with maximum score in 2021, 84.4% (54) were from the intervention group and 72.2% (65) from the control group, while in 2022, 80.0% (56) were from the intervention group and 79.3% (69) from control (Fig. 2B).

### Attitude about malaria prevention

**Fig. 3:**
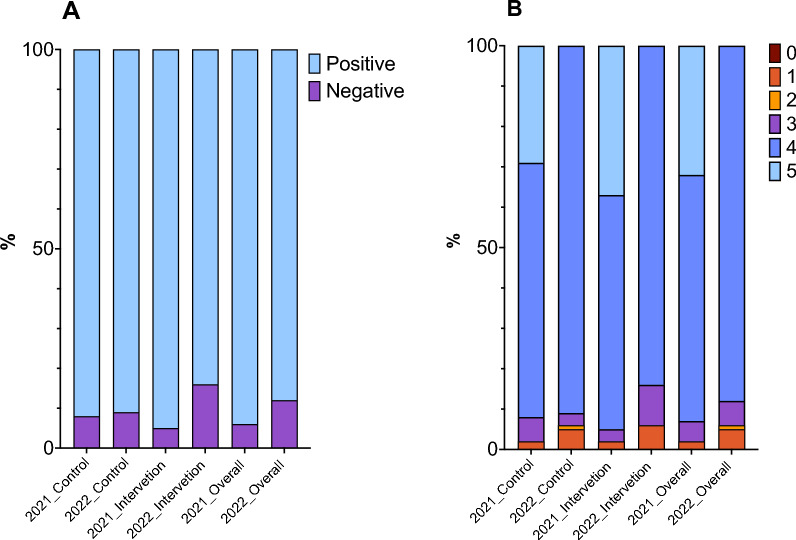
Attitude towards malaria prevention and their changes over time and intervention. **A** Categorized attitude. The light blue bars represent high attitude, and the purple bars represent low attitude. Positive attitudes correspond to the number of heads of households with attitude scores at or above the median (≥ 4 correct answers). Negative attitudes correspond to the number of heads of households with below median (4.0) number of correct answers. **B** Attitude scores. The brownish-red bars represent the number of participants with no scores, the dark yellow bars represent the number of participants with 1 score, the light yellow bars represents the number of participants with 2 scores, the purple bars represent the number of participants with 3 scores, the dark blue bars represent the number of participants with 4 scores, and the light blue bars represent the number of participants with 5 scores. Each score represents the number of correct answers, with higher scores reflecting more positive attitudes towards malaria prevention

High proportion of heads of households had positive attitudes about malaria prevention in 2021 [93.5% (144)] and in 2022 [87.9% (138)]. The attitudes of heads of households were also high between intervention and control in 2021, [95.3% (61), 92.2% (83)] and in 2022, [84.3% (59), 90.8% (79)] respectively (Fig. 3A). In 2021, 100.0% (154) of heads of households owned mosquito nets, 61.6% (93) recognized to be at risk of getting malaria, 96.0% (145) recognized that the use of mosquito net can prevent malaria, 100.0% (151) referred that malaria suspects should be taken to the health facility, and 99.3% (150) recognized malaria as a fatal disease. In 2022, 94.9% (149) heads of household owned mosquito net, 100.0% (149) were aware of being at risk of getting malaria, 89.9% (134) recognized that the use of mosquito net can prevent malaria, 100.0% (149) referred that if someone has malaria should be taken to the health facility, and 98.6% (145) recognize malaria as a fatal disease.

The overall attitude´s score in 2021 was 61.0% (94), 57.8% (37) from the intervention and 63.3% (57) from the control group. In 2022, the overall score for attitude was 87.9% (138), 84.3% (59) from intervention and 90.8% (79) from control (Fig. 3B).

### Practice related to malaria prevention

In 2021, 42.9% (63) used a mosquito net to prevent malaria, 96.1% (148) slept under the mosquito net in the previous night and 98.7% (149) mentioned to seek the health center when someone has malaria. In 2022, 47.3% (70) heads of households use a mosquito net to prevent malaria, 95.3% (142) slept under the mosquito net the previous night and 98.7% (147) referred that they seek a health facility when someone gets malaria.

**Fig. 4:**
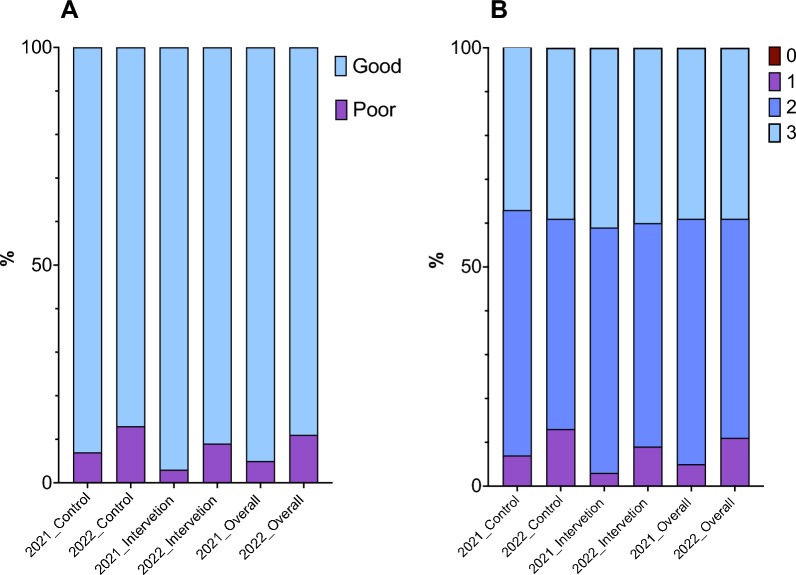
Comparison of malaria prevention practices. **A** Practice levels categorized by score. The light blue represents high practice, and the purple represents low practice. Good practice includes heads of households who answered correctly at or above the median score (≥ 2), while Poor practice includes those scoring below the median (< 2). **B** Attitude scores. The brownish red represents the number of participants with zero scores, the purple bars represent the number of participants with 1 score, the dark blue represents the number of participants with 2 scores, and the light blue represents the number of participants with 3 scores. Each score (1–5) represents the number of correct answers. Higher scores indicating better malaria prevention practice

More than 80% of heads of households had good practices regarding malaria prevention, in 2021 94.8% (146) and in 2022 89.2% (140). The practices in both, intervention and control group, were high in 2021 [96.9% (62), 93.3% (84)] and in 2022 [91.4% (64), 87.4% (76)], respectively (Fig. 4A).

Most heads of households had scored above 2 (out of 0–3). Approximately half of heads of households scored maximum in 2021 and 2022 [55.8% (86) and 49.7% (78) respectively]. (Fig. 4B).

### Association of KAP with sociodemographic and household characteristics in years 2021 and 2022

In 2021, before the intervention, the knowledge of signs, symptoms and malaria transmission (binary variable) was associated with ownership of mobile phone (p = 0.026) and a bicycle (p = 0,025). In 2022, the knowledge was associated with education level (p = 0.016) and marital status (p = 0.014).

In 2021, the attitudes towards malaria prevention (binary variable) were associated with education level (p = 0.025). In 2022, the attitudes towards malaria prevention were associated with mobile phone ownership (p =  < 0.001).

There were association of practice with the ownership of mobile phone (p = 0.008) and bicycle (p = 0.007) in 2021. In 2022 the practices have association with mosquito net (p = 0.005) and mobile phone ownership (p = 0.003) (S1 table sheet 2).

### Association of knowledge, attitude and practice scores with sociodemographic and household characteristics in years 2021 and 2022

When analyzed the association of knowledge scores with sociodemographic and household characteristic, no associations were found. In 2022 an association of knowledge with gender (p = 0.030) and mobile phone ownership (p = 0.021) was detected.

The attitudes towards malaria prevention were associated with marital status (p = 0.015) in 2021, while in 2022, they were associated with the ownership of mosquito bed net (p < 0.001).

In 2021, the practice related to malaria prevention were associated with occupation (p = 0.035), while in 2022, they were association with the ownership of mobile phone (p = 0.033).

### Association of knowledge, attitude and practices between intervention and control in years 2021 and 2022

No differences were found between households in the control and intervention groups in 2021 and 2022 in terms of KAP.

In 2021, before the stating of the project, there were associations between the knowledge of signs, symptoms and mode of transmission malaria with attitudes related to malaria prevention (p < 0.001) and practices of malaria prevention (p < 0.001) in 2021. In 2022 also were association between the knowledge of signs, symptoms and mode of malaria transmission with attitude regarding malaria prevention (p < 0.001) and practices of malaria prevention (p < 0.001) Table 1.

**Table 1:** Sociodemographic and households characteristics of the participants in before and after intervention

Variables	Before intervention						After intervention					
	Intervention		Control		Overall		Intervention		Control		Overall	
	N = 64		N = 90		N = 154		N = 70		N = 87		N = 157	
	%	N	%	N	%	N	%	N	%	N	%	N
Gender												
Male	51.6	33	55.6	50	53.9	83	51.4	36	47.1	41	49.0	77
Female	48.4	31	44.4	40	46.1	71	48.5	34	52.9	46	51.0	80
Age (median|IQR:25–75)	33.5 (24.8–50.5)						37.5(27.0–52.0)					
Age group												
18–25	26.6	17	26.7	24	26.6	41	22.9	16	19.5	17	21.0	33
26–35	25.0	16	26.7	24	26.0	40	22.9	16	27.6	24	25.5	40
≥ 36	48.4	31	46.7	42	47.4	73	54.3	38	52.9	46	53.5	84
Education												
No education	26.6	17	20.0	18	22.7	35	22.9	16	27.6	24	25.5	40
Primary	57.8	37	73.3	66	66.9	103	58.6	41	58.6	51	58.6	92
Secundary	15.6	10	6.7	6	10.4	16	18.6	13	13.8	12	15.9	25
Occupation												
Farmer	87.5	56	93.3	84	90.9	140	100.0	70	100.0	87	100.0	157
Other	12.5	8	6.7	6	9.1	14	0.0	0	0.0	0	0.0	0
Marital status												
Single	4.7	3	5.6	5	5.2	8	45.7	32	39.1	34	42.0	66
Married/marital union	76.6	49	75.6	68	76.0	117	42.9	30	42.5	37	42.7	67
Divorced	3.1	2	10.0	9	7.1	11	2.9	2	3.4	3	3.2	5
Widowed	15.6	10	8.9	8	11.7	18	8.6	6	14.9	13	12.1	19
Members per household (median|IQR:25–75)	5 (3.0–7.0)						5 (3.0–7.0)					
Ownership of mosquito net												
Yes	100.0	64	100.0	90	100.0	154	92.9	65	96.6	84	94.9	149
No	0.0	0	0.0	0	0.0	0	7.1	5	3.4	3	5.1	8
Mosquito net per household (median|IQR:25–75)	2(1.8–3.0)						2 (1.0–3.0)					
Number of ITNs per household												
0	0.0	0	0.0	0	0.0	0	1.5	1	0.0	0	0.7	1
1	25.0	16	24.4	22	24.7	38	24.6	16	38.1	32	32.2	48
2	37.5	24	27.8	25	31.8	49	33.8	22	29.8	25	31.5	47
3	15.6	10	34.4	31	26.6	41	24.6	16	22.6	19	23.5	35
4	15.6	10	10.0	9	12.3	19	9.2	6	7.1	6	8.1	12
5	3.1	2	1.1	1	1.9	3	1.5	1	2.4	2	2.0	3
6	3.1	2	2.2	2	2.6	4	0.0	0	0.0	0	0.0	0
7	0.0	0	0.0	0	0.0	0	1.5	1	0.0	0	0.7	1
8	0.0	0	0.0	0	0.0	0	1.5	1	0.0	0	0.7	1
10	0.0	0	0.0	0	0.0	0	1.5	1	0.0	0	0.7	1
Radio ownership												
Yes	26.6	17	23.3	21	24.7	38	28.6	20	28.7	25	28.7	45
No	73.4	47	76.7	69	75.3	116	71.4	50	71.3	62	71.3	112
Television ownership												
Yes	0.0	0	0.0	0	0.0	0	1.4	1	2.3	2	1.9	3
No	100.0	64	100.0	90	100.0	154	98.6	69	97.7	85	98.1	154
Mobilephone ownership												
Yes	62.5	40	56.7	51	59.1	91	60.0	42	57.5	50	58.6	92
No	37.5	24	43.3	39	40.9	63	40.0	28	42.5	37	41.4	65
Car ownership												
Yes	0.0	0	0.0	0	0.0	0	0.0	0	0.0	0	0.0	0
No	100.0	64	100.0	90	100.0	154	100.0	70	100.0	87	100.0	157
Motorcycle ownership												
Yes	7.8	5	8.9	8	8.4	13	10.0	7	12.6	11	11.5	18
No	92.2	59	91.1	82	91.6	141	90.0	63	87.4	76	88.5	139
Bicycle ownership												
Yes	65.6	42	55.6	50	59.7	92	58.6	41	60.9	53	59.9	94
No	34.4	22	44.4	40	40.3	62	41.4	29	39.1	34	40.1	63
Water source ownership at home												
Yes	3.1	2	5.6	5	4.5	7	50.0	35	54.0	47	52.2	82
No	96.9	62	94.4	85	95.5	147	50.0	35	46.0	40	47.8	75
Main Source of water supply												
Standpipe	84.4	54	74.4	67	78.6	121	67.1	47	60.9	53	63.7	100
Well water	15.6	10	25.6	23	21.4	33	25.7	18	31.0	27	28.7	45
River water	0.0	0	0.0	0	0.0	0	7.1	5	6.9	6	7.0	11
Neighborhood	0.0	0	1.1	1	0.6	1	0.0	0	0.0	0	0.0	0
Lake	0.0	0	0.0	0	0.0	0	0.0	0	1.1	1	0.6	1
Power source ownership in the main build												
Yes	0.0	0	0.0	0	0.0	0	0.0	0	0.0	0	0.0	0
No	100.0	64	100.0	90	100.0	154	100.0	70	100.0	87	100.0	157
Firewood as the main power source for prepare meal												
Yes	100.0	64	100.0	90	100.0	154	100.0	70	100.0	87	100.0	157
No	0.0	0	0.0	0	0.0	0	0.0	0	0.0	0	0.0	0
Ownership of animals in the household												
Yes	79.7	51	68.9	62	73.4	113	78.6	55	81.6	71	80.3	126
No	20.3	13	31.1	28	26.6	41	21.4	15	18.4	16	19.7	31

When comparing the answers from heads of households from the intervention or control groups in 2021 and 2022, no significance in knowledge attitudes and practices were observed (S1 table sheet 3).

### Factors associated with knowledge, attitude and practices of the participants

In 2021, the high knowledge was associated with good practices (OR = 0.012 95% CI 0.001–0.132). In 2022, the high knowledge was associated with positive attitudes (OR: 0.010 95% CI 0.001–0.091) and good practices (OR: 0.006 95% CI 0.001–0.059). In 2022, good practices were associated with the ownership of mosquito bed net (p = 0.009) and mobile phone (OR = 0.190, P = 0.015) (Table 2).

**Table 2:** Factors associated with knowledge, attitude and practices of the participants before and after intervention

	Before intervention		After intervention	
Variable	OR	p-value	OR	p-value
Education				
No education	Ref	–	–	–
Primary	3.322	0.155	–	–
Secundary	216,143,262	0.998	–	–
Marital status				
Single	7,711,817.769	0.999	–	–
Married/marital union	0.326	0.362	–	–
Divorced	0.100	0.113	–	–
Widowed	Ref	–	–	–
Ownership of mosquito net				
Yes	–	–	0.115	0.009
No	Ref	–	–	–
Ownership mobile phone				
Yes	0.190	0.160	0.217	0.015
No	Ref	–	Ref	–
Ownership of radio				
Yes	–	–	0.503	0.396
No	–	–	–	–
Ownership of bycicle				
Yes	0.228	0.205	–	–
No	Ref	–	–	–

## Discussion

This study assessed knowledge, attitudes and practices regarding malaria, among Tambai community, to understand whether the higher burden of malaria was related to knowledge, attitude and practices towards malaria. The findings indicate high knowledge of malaria both in 2021 and 2022. These high knowledge have been described in comparable KAP studies in Africa (South Africa[23], Nigeria[24]), Afghanistan[25] and Colombia[26]. The association of mosquito bite with malaria transmission was reported by the majority of household heads interviewed in the study, revealing specific knowledge of malaria transmission, which has also been observed in other rural malaria endemic areas in Asia [27] and South Africa[23].

Looking into malaria prevention, more than 80% of heads of households mentioned sleeping under a mosquito bed net as main malaria preventive measure. This result is not consistent with the study implemented in a high incidence urban area of Santo Domingo, Dominican Republic, where less than half of participants indicated any correct malaria preventive measure[28]. These differences might be associated with particular community characteristics or broader features like rural/urban factors[29]. Artemisinin and lumefantrine, known as Coartem™ was mentioned as malaria treatment by most of household heads, contrasting with the knowledge, attitude and practice study held in South Africa where the participants had low knowledge of malaria treatment [23].

The overall knowledge observed was high and similar for intervention and control groups, the slight reduction observed on intervention and control groups, in 2022 can partially be explained by changes in heads of households between the initial and the last KAP study.

The lack of significant difference between intervention and control groups may also be explained by frequent presence of healthcare professionals, as part of the routine community health services provided by integrated health teams from district health authorities. The routine community health services provided by integrated teams of health providers are designed to bring health services to the most peripheral and remote populations. They offer different services such as lectures, screening services, nutrition services, antenatal consultation, malaria diagnosis and treatment. In addition, the project (REPELMALARIA) held monthly meetings to raise public awareness about malaria prevention. Although knowledge of symptoms, mode of malaria transmission, prevention and treatment of malaria was high in 2021 and in 2022, the burden of malaria remains high in the Tambai community. A recent study aimed to map mosquitos breeding sites and determining malaria prevalence in the same area shown that malaria prevalence remains high[30].

Mobile phone and bicycles, a proxy of economic wellness among the community presented an association with high knowledge, positive attitudes and good practices. In 2022, there were association between mosquito net ownership and good practices. Previous studies have associated higher wealth and education levels with positive attitudes towards malaria prevention, wealthier adolescents demonstrated better malaria prevention practices[9]. Another, community-based KAP study conducted among households in Makenene, Centre-Cameroon, found that being a public servant was associated with better malaria control practices. Furthermore, public servants were also more likely to exhibit positive attitudes, therefore, suggesting an association between occupation and wealth as favorable attitudes toward malaria treatment [31].

The high knowledge, attitude and practice within the population in 2021 contributed to the high level observed in 2022. Thus, the importance of permanent lectures regarding malaria and other disease prevention and control within the community. A similar effect was observed in another KAP study conducted in Dakar, Senegal which found high knowledge, attitude and practice before and post intervention, without detecting particular effect attributable to the intervention itself [11].

The variations of householders within the same household led to different individuals being interviewed in both years. This may have contributed to the low number of significant associations observed. Additionally, the intervention in Tambai was not focused on health promotion or education, which may explain the contrasting findings described by the study of [32], which reported a significant increase in knowledge and attitude in the intervention group.

During the implementation period the household varied, due to migration from and to the study area, this might come as a limitation a paired analysis could lead to more robust information.

The knowledge, attitude and practice about malaria was high at pre- and post- intervention, even so, the malaria burden remain high in the study area, suggesting the need for future studies to investigate the reasons behind this apparent discrepancy. This would be useful, to design interventions that goes behind information on malaria, engaging the population to transform the current KAP into local activities to stop malaria transmission and increase community-proximity to diagnosis and treatment.

## Conclusion

The knowledge, attitude and practice about symptoms, mode of transmission, prevention and treatment of malaria among the heads of households of Tambai were high in 2021 and 2022. Both intervention and control groups showed high KAP, suggesting that the implementation of the REPELMALARIA project did not have an either positive nor negative impact on KAP scores. Therefore, these findings suggests that higher malaria cases in the study area are not directedly related to knowledge, attitude and practice from the householders. Even so, continuous awareness-raising lectures on malaria prevention carried out by the REPELMALARIA project in collaboration with the Nhamatanda district health authority most likely contributed to sustain the community’s high levels of knowledge, attitudes, and practices (KAP) observed after one year of intervention. The community knowledge was significantly associated with good practices and positive attitudes. Despite the apparent low impact of KAP, on malaria burden as detected in the study. It is recommend that malaria programmes continue delivering educational lectures in remote communities, particularly those difficult to access, with limited resources and far from health facilities.

## Supplementary Information

Supplementary Material 1. Table 2. Association of Knowledge, Attitude and Practice about symptoms, transmission mode, preventive measure of malaria before and after, intervention and control. Table 3. Association of knowledge, attitude and practices between intervention and control before and after intervention

## Data Availability

Data is provided within the manuscript and supplementary information files.
